# Electro-Elastic Instability and Turbulence in Electro-osmotic Flows of Viscoelastic Fluids: Current Status and Future Directions

**DOI:** 10.3390/mi16020187

**Published:** 2025-02-04

**Authors:** Chandi Sasmal

**Affiliations:** Department of Chemical Engineering, Indian Institute of Technology Ropar, Rupnagar 140001, India; csasmal@iitrpr.ac.in

**Keywords:** viscoelastic fluids, electrokinetic flows, electro-elastic instability, electro-elastic turbulence

## Abstract

The addition of even minute amounts of solid polymers, measured in parts per million (ppm), into a simple Newtonian fluid like water significantly alters the flow behavior of the resulting polymer solutions due to the introduction of fluid viscoelasticity. This viscoelastic behavior, which arises due to the stretching and relaxation phenomena of polymer molecules, leads to complex flow dynamics that are starkly different from those seen in simple Newtonian fluids under the same conditions. In addition to polymer solutions, many other fluids, routinely used in various industries and our daily lives, exhibit viscoelastic properties, including emulsions; foams; suspensions; biological fluids such as blood, saliva, and cerebrospinal fluid; and suspensions of biomolecules like DNA and proteins. In various microfluidic platforms, these viscoelastic fluids are often transported using electro-osmotic flows (EOFs), where an electric field is applied to control fluid movement. This method provides more precise and accurate flow control compared to pressure-driven techniques. However, several experimental and numerical studies have shown that when either the applied electric field strength or the fluid elasticity exceeds a critical threshold, the flow in these viscoelastic fluids becomes unstable and asymmetric due to the development of electro-elastic instability (EEI). These instabilities are driven by the normal elastic stresses in viscoelastic fluids and are not observed in Newtonian fluids under the same conditions, where the flow remains steady and symmetric. As the electric field strength or fluid elasticity is further increased, these instabilities can transition into a more chaotic and turbulent-like flow state, referred to as electro-elastic turbulence (EET). This article comprehensively reviews the existing literature on these EEI and EET phenomena, summarizing key findings from both experimental and numerical studies. Additionally, this article presents a detailed discussion of future research directions, emphasizing the need for further investigations to fully understand and harness the potential of EEI and EET in various practical applications, particularly in microscale flow systems where better flow control and increased transport rates are essential.

## 1. Introduction

Electrokinetic (EK) flow refers to the movement of a fluid containing charged ions or particles suspended in a fluid, induced by an applied electric field [1]. EK flows can be broadly classified into two main types: electro-osmosis, which involves the bulk motion of a conductive fluid relative to a stationary charged surface, and electrophoresis, which refers to the motion of charged particles relative to the surrounding fluid. In addition to these, other forms of EK flows include diffusiophoresis, sedimentation potential, streaming potential, and streaming current. These electrokinetic phenomena are pivotal in a wide range of applications due to their ability to precisely manipulate fluids and particles at micro- and nanoscales, making them particularly useful in fields such as microfluidics, analytical chemistry, environmental engineering, and biomedicine. For instance, EK flows play a crucial role in microfluidic systems and lab-on-a-chip devices, where they are employed in applications such as DNA sequencing, cell sorting, and performing a variety of biochemical reactions with high precision [2,3,4]. In capillary electrophoresis, EK flows are used to separate ions or molecules based on their size and charge, enabling the high-resolution analysis of complex mixtures in chemistry and biology [5,6,7]. In environmental engineering, EK remediation techniques are utilized to extract and remove contaminants such as heavy metals, organic compounds, and other pollutants from soils and sediments [8,9,10]. Furthermore, in biomedical applications, EK flows facilitate the controlled and precise transport of drugs, enabling targeted drug delivery in microfluidic devices [11,12,13]. EK flows are also critical in fuel cell technology, particularly in proton exchange membrane (PEM) fuel cells, where they facilitate the transport of water and ions, enhancing the efficiency of the system [14,15]. The wide range of EK flow applications, especially in small-scale systems like microfluidics and lab-on-a-chip devices, stems from their advantages over traditional pressure-driven flows. Unlike pressure-driven flows, EK flows enable the more precise control of small fluid volumes, exhibit less flow resistance due to their plug-like velocity profile (as opposed to the parabolic profile seen in pressure-driven systems), and do not rely on mechanical moving parts [16]. This lack of mechanical moving components simplifies the design and operation of systems, making EK flows more suitable for applications in microscale systems, where traditional pressure-driven flows may become challenging to operate and accurately control.

The flow dynamics during electrokinetic flows are influenced by a variety of factors, including the strength of the imposed electric field, the type of electric field (AC or DC), the magnitude of the wall zeta potential, the geometry of the system, the presence of rotating or non-rotating surfaces, and the inclusion of obstacles within the flow path [1,16,17]. Over the past several decades, numerous studies have explored how these parameters affect EK flow behavior, leading to a deeper understanding of flow control and optimization. In addition to these physical and geometric factors, the rheological properties of the fluid itself, particularly when dealing with non-Newtonian fluids, play a critical role in influencing EK flow dynamics. A wide range of non-Newtonian or complex fluids, such as polymer solutions, emulsions, suspensions, and foams, are routinely used in microfluidic applications [18,19,20]. Moreover, biofluids like blood, saliva, cerebrospinal fluid, and suspensions of biomolecules such as DNA and proteins are frequently encountered in microfluidic and nanofluidic processes, particularly in diagnostics and biochemical analyses under EK flow conditions [21,22,23,24]. Unlike Newtonian fluids, which follow the simple linear relationship between the applied force and fluid deformation described by Newton’s law of viscosity, non-Newtonian fluids exhibit more complex non-linear behaviors, such as shear-thinning, shear-thickening, and yield stress. Furthermore, many of these non-Newtonian fluids possess viscoelastic properties, where the fluid exhibits both viscous and elastic behaviors depending on the flow conditions and applied stresses [25]. This viscoelasticity, combined with other non-Newtonian properties, introduces significant complexity to the EK flow dynamics. In particular, the interaction between the fluid’s microstructure and the electric field often leads to behaviors that deviate considerably from those seen in Newtonian fluids under similar conditions. As a result, understanding and predicting EK flow in non-Newtonian fluids requires special attention from researchers, which remains a critical and evolving area of research with wide-reaching implications.

In particular, the flow dynamics of viscoelastic fluids under an electric field warrant special attention due to the complex and intricate flow physics that emerge during their flow. This viscoelastic behavior arises from changes in the molecular conformation of microstructures within the fluid, such as polymer or surfactant molecules, which undergo stretching and relaxation as they respond to deformation in the flow field [26,27]. Similar behavior can be observed in fluids containing naturally occurring microstructures, such as red blood cells in blood [23,28], droplets in emulsions [29,30], bubbles in foams [31,32], and particles in suspensions [33,34]. These microstructures experience stretching and subsequent relaxation under deformation, contributing to the fluid’s viscoelasticity. To quantify the degree of elasticity in viscoelastic fluids, researchers typically use either the Weissenberg number (Wi) or the Deborah number (De). Both these dimensionless numbers express the relationship between the fluid’s microstructural relaxation time (for instance, the relaxation time λ of polymer molecules in polymer solutions) and a characteristic time scale of the flow. However, the two parameters differ slightly in characterizing this time scale. For the Weissenberg number, the characteristic time scale is defined indirectly, typically using the inverse of the flow deformation rate or shear rate $\left(\dot{\gamma }\right)$, expressed as $Wi=\lambda \dot{\gamma }$. In contrast, the Deborah number is based on the time *T* a fluid element takes to travel a certain distance in a Lagrangian sense, and it is defined as $De=\frac{\lambda }{T}$. Although the expressions for Wi and De are different, they can become identical or related with a geometrical factor depending on the specific flow configuration and conditions. For example, in the case of flow through a square cavity with height and width *H*, both the deformation and travel time scales are proportional to $\frac{H}{U_{ch}}$, where $U_{ch}$ is the characteristic fluid velocity. In this scenario, the expressions for both the Weissenberg and Deborah numbers are identical, i.e., $Wi=De=\frac{\lambda U_{ch}}{H}$. However, in a flow through a rectangular cavity with height *H* and width *L*, the time scales differ due to the geometry, leading to a relationship between the Weissenberg and Deborah numbers that incorporates a geometric factor of $\frac{L}{H}$, i.e., $Wi=De\frac{L}{H}$. Another two dimensionless numbers are relevant for investigating the flow dynamics of viscoelastic fluids in microscale systems, namely, the Reynolds number (Re) and the elasticity number (El). The former dimensionless number signifies the ratio of inertial forces to viscous forces, which generally remains less than one for microscale flows due to the small sizes of the system. The second number is the ratio of the Weissenberg and Reynolds numbers, i.e., $El=\frac{Wi}{Re}$. High values of this ratio suggest that elastic forces are dominant over inertial forces, whereas low values denote the opposite [35].

When this dimensionless Weissenberg or Deborah number exceeds a critical threshold (or the elasticity number becomes very high), a transition from stable to unstable flow often occurs in viscoelastic fluids during their flow. This flow transition is triggered when the elastic stresses in the viscoelastic fluid interact with the curvature of the streamlines, especially in small-scale systems where inertial forces are negligible. Therefore, this type of flow instability is purely driven by the elastic stresses of the fluid and is termed elastic instability (EI). Elastic instabilities were initially studied in rotational shear flows, such as concentric plates as well as plate or cone and plate geometries. These geometries are widely used in rheometry to measure fluids’ rheological properties. For instance, Phan-Thien [36,37] demonstrated through linear stability analysis that flow becomes unstable when the Weissenberg number, defined as Wi=λΩ (where Ω is the angular velocity of rotation), exceeds a critical value in both concentric plate, plate, and cone and plate configurations. These theoretical predictions were among the first to establish the conditions under which viscoelastic fluids experience flow instability. The first comprehensive experimental and theoretical study of elastic instabilities in rotational shear flows was conducted by Larson et al. [38]. Focusing on the Taylor–Couette flow system (a flow between two concentric cylinders), they used linear stability theory for an Oldroyd-B viscoelastic fluid to predict the onset of elastic instabilities. Their analysis showed that a critical Deborah number of the form $f\left(s\right)\epsilon^{-\frac{1}{2}}$ governs the instability threshold, where f(s) is a function of the ratio of solvent to polymer contributions to the solution’s viscosity, and ϵ is the ratio of the gap between the cylinders to the radius of the inner cylinder. Above this critical Deborah number value, the flow transited to a growing oscillatory flow, which was seen both in their theoretical analysis and experiments. The study was not only groundbreaking for its agreement between theory and experiment but also provided a detailed mechanism for the onset of elastic instabilities. Following Larson et al.’s [38] seminal work, extensive research has investigated the origins and mechanisms of elastic instabilities across various flow geometries [39,40]. As the Weissenberg or Deborah number continues to increase beyond the point of elastic instability, the flow transitions to a more chaotic and turbulent-like state, known as elastic turbulence (ET). Unlike classical turbulence, which is driven by inertial forces, elastic turbulence arises from the complex interactions between the elastic forces and flow deformation, leading to chaotic and time-dependent flow patterns. Therefore, elastic instability serves as the precursor to elastic turbulence. Several review articles have provided comprehensive insights into elastic instability and elastic turbulence phenomena, particularly in the pressure-driven flows of viscoelastic fluids. These reviews highlight the significant progress in understanding the fundamental mechanisms underlying these flow transitions and their implications for various applications [40,41,42].

Compared to the extensive body of research on elastic instability and elastic turbulence in the pressure-driven flows of viscoelastic fluids, investigations into these phenomena in electrokinetically driven flows remain limited. In electrokinetic-driven systems, these phenomena are referred to as electro-elastic instability (EEI) and electro-elastic turbulence (EET) to emphasize their distinct origin compared to pressure-driven counterparts. Despite the differences in driving forces, i.e., whether pressure or electrokinetically driven, the potential of both EI and ET to enhance transport processes such as mixing, heat, and mass transfer in microscale systems is immense. In microfluidic systems, where transport rates are often dominated by slow molecular diffusion, the chaotic and unstable flow fields generated by these instabilities can dramatically increase convective transport rates, even under low Reynolds numbers or creeping flow conditions. This enhancement in transport processes has been well established in pressure-driven EI and ET through both experimental and numerical studies, particularly in areas like microscale mixing [43,44,45] and heat transfer [46,47,48]. However, similar investigations on electrokinetically driven flows are notably scarce. While pressure-driven EI and ET have been explored extensively, both for their potential practical applications and fundamental understanding, research on EEI and EET is still in its infancy. As a result, there is a significant gap in understanding how these phenomena operate under electrokinetic flow conditions, where the interaction between fluid viscoelasticity and electric fields may lead to unique flow behaviors.

This article aims to bridge this gap by offering a detailed exploration of the future directions for research on EEI and EET, considering both practical applications and fundamental understanding. We also provide a comprehensive review of the limited studies available on electrokinetic-driven EI and ET (particularly electro-osmotic-driven EI and ET), highlighting the critical areas where further research is needed. By identifying these gaps in the literature, we aim to encourage more in-depth investigations into the mechanisms of electro-elastic instabilities and turbulence, as well as their potential applications in various fields.

## 2. Current Status of Studies on EEI and EET

**Figure 1:**
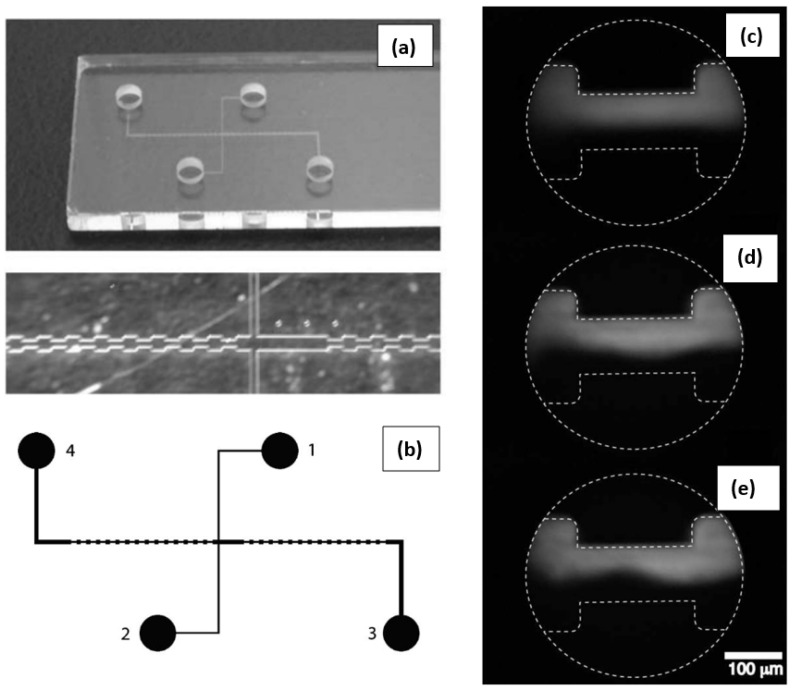
(**a**,**b**) The microfluidic geometry used in the experimental studies of Bryce and Freeman [49,50], consisting of a microchannel with step expansion and contraction (in a 2:1 ratio) for the electro-osmotic transport of both simple Newtonian (methanol and water mixture) and viscoelastic (polyacrylamide dissolved in methanol and water mixture) fluids. The microfluidic setup had four reservoirs: two of them (Reservoirs 1 and 2) were used to push dyed and undyed fluids into the main microchannel, and, ultimately, they were collected in Reservoir 4. The interface between dyed and undyed fluids in the cases when they were Newtonian fluids (**c**) and viscoelastic fluids at two different time instances (**d**,**e**) at the same applied voltage of 0.2 kV.

Bryce and Freeman [49,50] were among the first to experimentally demonstrate the existence of electro-elastic instability during the flow of viscoelastic polymer solutions. These solutions were prepared using polyacrylamide (PAA) polymers dissolved in a 20:80 vol.% methanol mixture, and the experiments were conducted in a microfluidic expansion and contraction geometry with a 2:1 ratio, as shown in Figure 1a,b. The experimental setup involved two fluid streams: a dyed viscoelastic fluid (colored with tetramethylrhodamine dye) entered the microfluidic device from Reservoir 1, while an undyed viscoelastic fluid entered from Reservoir 2. The two streams merged and flowed through the expansion–contraction microchannel before being collected in Reservoir 4. Figure 1c–e illustrates the interface behavior between the dyed and undyed fluids for both simple Newtonian fluids (polymer-free solvents) and viscoelastic fluids under an imposed electric voltage of 0.2 kV. In the case of the Newtonian fluids, the interface remained stable and nearly straight, as expected in a steady, laminar flow regime typical of creeping flow conditions for Newtonian fluids, as shown in Figure 1c. However, for viscoelastic fluids, the interface became unstable and showed fluctuations, demonstrating the onset of electro-elastic instability. These fluctuations were time-dependent, as indicated by their varying appearances at different time instances, as shown in Figure 1d,e. The researchers also observed that the fluctuation intensity of the interface increased with the applied voltage, showing a direct relationship between the electric field strength and the instability intensity. Interestingly, the behavior of the interface with respect to polymer concentration followed a different trend: initially, the fluctuation intensity increased with polymer concentration, but after exceeding a critical concentration (below the polymer overlap concentration), the fluctuation intensity plateaued or slightly decreased. Despite the generation of EEIs within the microfluidic device, the mixing behavior of the dyed and undyed viscoelastic fluids was found to decrease compared to that of Newtonian fluids. This reduction in mixing was counterintuitive, as this instability typically leads to enhanced mixing in pressure-driven elastic instabilities and elastic turbulence, where viscoelastic fluids generally show much higher mixing efficiencies than Newtonian fluids. Bryce and Freeman proposed two reasons for this reduced mixing behavior. First, they hypothesized that the large-scale fluctuations generated by the electro-elastic instabilities might not effectively mix the fluids at the smaller molecular scale, which is critical for efficient mixing. Second, they suggested that the absence of shear deformation in the microfluidic device, which is crucial for mixing, plays a role. Instead, they anticipated that the flow inside the device is dominated by extensional deformation, which is less conducive to mixing than shear deformation. This finding contrasts sharply with the behavior of pressure-driven elastic instabilities, where the mixing efficiency typically increases significantly in viscoelastic fluids. These results highlight the complex interplay between electro-elastic instabilities and the underlying flow mechanisms, suggesting that additional factors, such as deformation modes, may play a critical role in determining the mixing efficiency in such systems.

**Figure 2:**
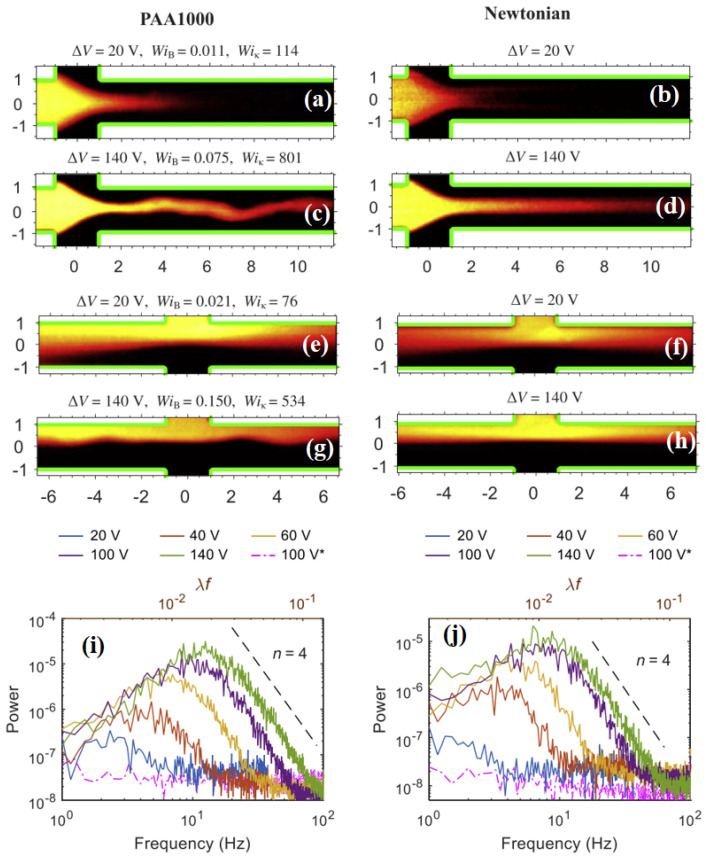
Interface patterns between dyed (bright color) and undyed (black color) fluids for viscoelastic polyacrylamide polymer solutions (PAA1000) and a Newtonian fluid (water) at two different applied voltages, namely, 20 V and 140 V, for a cross-shaped microchannel [52]. The results are shown here for two microchannel configurations, namely, flow-focusing (**a**–**d**) and cross-slot (**e**–**h**). Subfigures in the last row represent the power spectrum of the temporal variation in the interface position at different applied voltages for the flow-focusing (**i**) and cross-slot (**j**) configurations of the cross-shaped microchannel. Here, $Wi_{B}$ and $Wi_{\kappa }$ are the bulk and EDL Weissenberg numbers, respectively, defined based on the bulk shear rate within the geometry and the shear rate within the EDL, respectively.

Next, Afonso et al. [51] developed a numerical platform based on the finite volume method (FVM) for simulating the EEI phenomenon originating in a microfluidic cross-slot cell, which is widely used for studying and examining viscoelastic instabilities in a mixed flow environment, i.e., having both shear- and extension-flow-dominated regions inside it. They used the upper convected Maxwell (UCM) and simplified Phan–Thien–Tanner (sPTT) constitutive models to represent the viscoelastic rheological behaviors of the fluid. Their study revealed that when the dimensionless Weissenberg number (defined as $Wi=\frac{\lambda u_{sh}}{H}$, where λ is the polymer molecule relaxation time, $u_{sh}$ is the Helmholtz–Smoluchowski electro-osmotic velocity [16], and *H* is the channel width) exceeds a critical value, the flow field inside the cross-slot cell transitions from a steady and symmetric state to an unsteady and asymmetric flow state, indicating the origin of the electro-elastic instabilities inside it. This critical value of the Weissenberg number was again found to decrease with increasing electrical double-layer (EDL) thickness inside the cross-slot cell. They showed that the origin of this flow transition is the corners of the cross-slot (where shear flow is mainly dominant), where a strong interaction happens between the elastic stresses and streamline curvature present in this region. On the other hand, Pimenta and Alves [52] conducted both experimental and numerical investigations on the electro-osmotic flows of both viscoelastic PAA polymer solutions and water in a cross-shaped microchannel under different applied voltage conditions. Two configurations of the microchannel were considered, namely, flow-focusing and cross-slot configurations. In the first configuration, fluids enter the geometry through three inlets and leave through one outlet, whereas in the second configuration, fluids enter the geometry through two inlets and leave through two outlets. Figure 2 demonstrates the interface patterns between the dyed and undyed fluids at two different applied voltage conditions both for viscoelastic and Newtonian fluids. At a low applied voltage, i.e., at 20 V, both the viscoelastic and Newtonian fluids exhibited straight and stable interface patterns for both flow-focusing (a,b) and cross-slot (e,f) microchannel configurations. However, as the voltage increased to 140 V, the viscoelastic fluids displayed an unstable and wavy interface pattern, irrespective of the microchannel configuration (see (c) and (d) for flow-focusing and (g) and (h) for cross-slot configurations). In contrast, the Newtonian fluids still exhibited the same stable and straight interface pattern for both microchannel configurations as observed at 20 V. This suggests that the dye interface pattern starts to fluctuate once the applied voltage exceeds a critical value in the case of viscoelastic fluids due to electro-elastic instability. They further analyzed these temporal fluctuations in dye interface patterns at different applied voltage values by calculating their power spectrum, as presented in Figure 2i,j for the flow-focusing and cross-slot configurations, respectively. From these subfigures, it is not possible to identify individual power peaks at defined frequencies, suggesting the nature of fluctuation to be very random. As the imposed voltage increased, the fluctuation intensity increased, covering a wide range of excitation frequencies, regardless of the microchannel configuration. The power spectra exhibited a power-law decay $\left(P\propto f^{-n}\right)$ within the mid- to high-frequency range, spanning almost one decade of frequencies for the higher voltages. After the critical voltage (and/or the Weissenberg number) exceeded the critical value, the power-law decay spectral index remained almost constant (with a value of n=4) and independent of the applied voltage. In the case of rotational shearing viscoelastic fluid flows, Groisman and Steinberg [53,54] also experimentally found the excitation of fluctuation over a wide range of length and time scales, and the power spectra of fluctuation also exhibited a power-law decay over a wide range of frequencies. The power-law exponent value of n=3.5 was found in their study, and the corresponding flow regime was termed as the elastic turbulence regime. This value of *n* in the elastic turbulence regime was also predicted theoretically [55,56]. Later, many studies on pressure-driven flows of viscoelastic fluids through various geometries found the same value of *n* and confirmed the existence of elastic turbulence in those flows [57,58,59,60]. Pimenta and Alves [52] also observed a value of n=4, which was larger than predicted for the elastic turbulence regime for pressure-driven flows. However, this value was obtained from the time-series analysis of the dye interface fluctuation, which was not a fixed point data used for predicting the value if *n* in the case of pressure-driven elastic turbulent flows. Therefore, they were unsure whether their flow field transitioned to an electro-elastic turbulent regime. This uncertainty also came from the fact that the mixing behavior of dyed and undyed viscoelastic fluids was not improved up to the extent expected as if an elastic turbulent flow regime was present. Their corresponding numerical study based on the finite volume method (FVM) discretization technique provided further insights and mechanisms behind the origin of the EEI phenomenon. In particular, they found that the corners for both microchannel configurations are more susceptible to the origin of these instabilities due to the high streamline curvature and elastic stresses present in these regions. Sadek et al. [61] conducted a study comprising both experiments on and numerical simulations of electro-osmotic flows of viscoelastic fluids through several microchannel configurations such as microchannels with hyperbolic contraction, then sudden expansion (designated as forward configuration) or sudden contraction, and then hyperbolic expansion (designated as backward configuration). They also observed a transition in the flow field from steady and symmetric to unsteady and asymmetric, and finally irregular and chaotic due to the EEI phenomenon as the applied voltage difference gradually increased. However, they found that the intensity of the EEI phenomenon was larger for the backward configuration than for the forward one.

**Figure 3:**
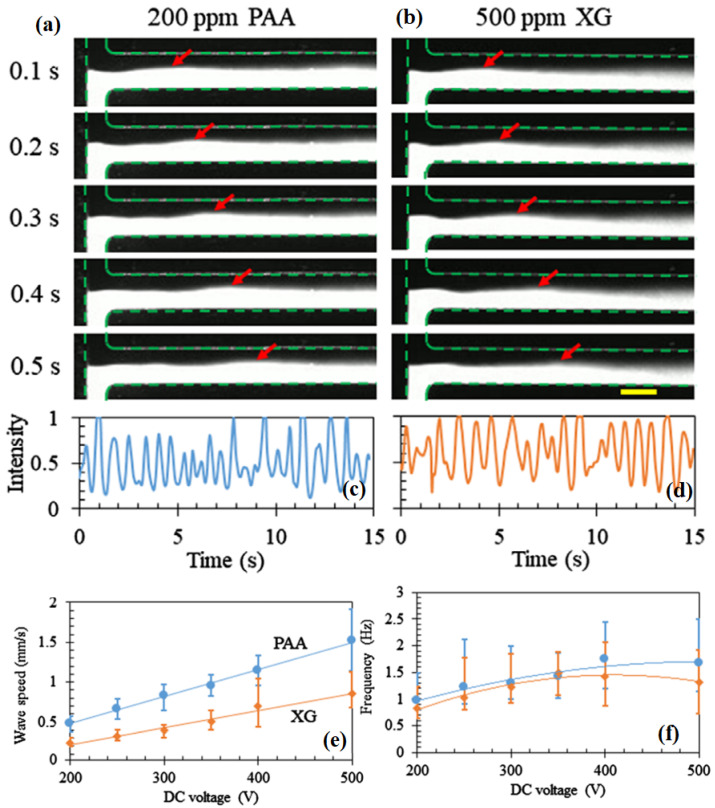
Electro-osmotic flows of polyacrylamide and xantham gum (XG) polymer solutions through a T-shaped microchannel [62] at an imposed voltage of 400 V. Subfigures (**a**,**b**) show the sequences of images demonstrating the propagation of waves (whose front is marked with the red arrow) generated due to the electro-elastic instability in PAA and XG solutions, respectively. The temporal variations in the dye interface pattern for these two solutions are shown in subfigures (**c**,**d**), whereas the corresponding wave speed and frequency of these fluctuations are shown in subfigures (**e**,**f**).

Song et al. [62] conducted an experimental study on the electro-osmotic flows of various polymer solutions through a T-shaped microchannel at different applied voltages. They also observed that the interface between dyed and undyed fluids oscillates once the imposed voltage exceeds a critical value due to the appearance of electro-elastic instability in this geometry; likewise, Pimenta and Alves [52] observed this in a cross-shaped microchannel. This is demonstrated in Figure 3a,b for polyacrylamide and xantham gum polymer solutions, which were elastic as well as highly shear-thinning in nature. The oscillation of this dye interface eventually created a wave whose front gradually traveled from the T junction of the microchannel toward its outlet, as shown by red arrows in these subfigures. The temporal fluctuations in the dye interface in the PAA and XG polymer solutions arising due to the EEI phenomenon are shown in Figure 3c and d, respectively. They further analyzed these temporal fluctuations at different imposed voltages. They calculated the speed at which the wave propagated and the frequency of the wave, as presented in Figure 3e,f for the two polymer solutions. It can be seen that the wave speed gradually increased with the applied voltage, whereas the frequency seemed to plateau at higher voltages for both polymer solutions. They concluded that the fluid elasticity and shear-thinning properties together might facilitate the generation of the EEI phenomenon rather than only the elastic properties of a polymer solution, as they did not observe the EEI phenomenon in polymer solutions, such as in polyethylene oxide (PEO), polyvinylpyrrolidone (PVP), and hyaluronic acid (HA), that were elastic but weakly shear-thinning.

**Figure 4:**
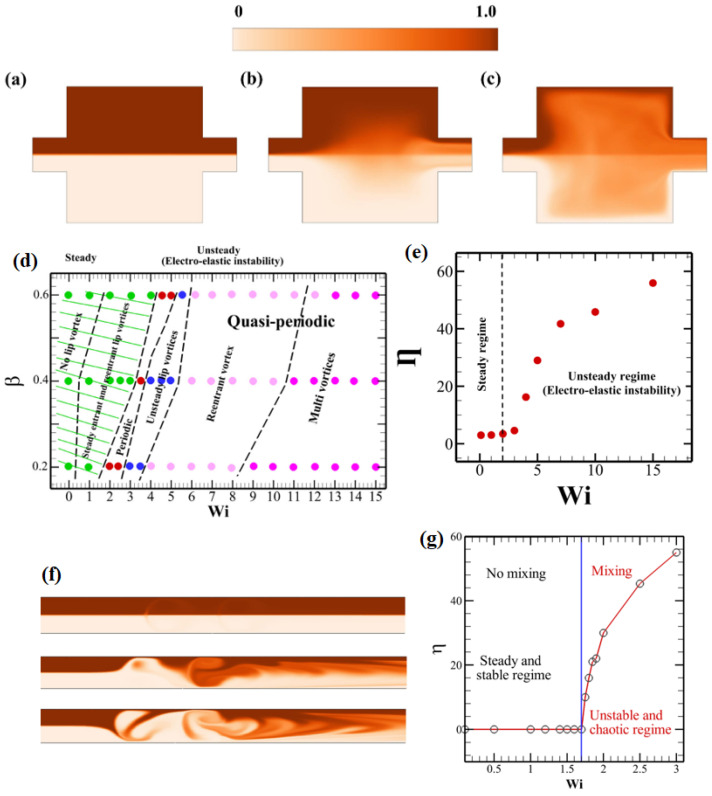
Electro-osmotic flows of viscoelastic fluids through a long microchannel with step expansion and contraction [63]. The time-averaged dye concentration profiles inside the microchannel at three different Weissenberg numbers, namely, 0.1 (**a**), 6 (**b**), and 15 (**c**). Here, the top and bottom halves of the microchannel were filled with dyed and undyed fluids, respectively. (**d**) A phase diagram showing the existence of different flow states and vortex structures inside the geometry in Wi−β space. (**e**) The mixing efficiency parameter η variation with the Weissenberg number inside the microchannel with step expansion and contraction. (**f**) The instantaneous dye concentration profiles and (**g**) mixing efficiency parameter η inside a microchannel with heterogeneous wall zeta potential at different Weissenber numbers [64].

Recently, Khan and Sasmal [63] performed a numerical study on the electro-osmotic flows of viscoelastic fluids through a long microchannel with step expansion and contraction present in it. The simulations were performed using the Oldroyd-B viscoelastic constitutive relation over a wide range of parameters like the Weissenberg number and polymer viscosity ratio. In particular, their study used a constant shear viscosity elastic fluid and/or a Boger fluid to investigate the explicit role of fluid elasticity on the generation of electro-elastic instability. They also investigated how this EEI phenomenon ultimately influenced the mixing behavior of the two fluids inside this geometry. Figure 4a–c depict the dye concentration variation inside the geometry, wherein the upper half of the geometry was a dyed fluid, whereas the bottom half was an undyed fluid at three different Weissenberg numbers, namely, 0.1 (a), 6 (b), and 15 (c). At a low Weissenberg number, i.e., at Wi=0.1, a stable and straight dye interface was seen (Figure 4a), and no mixing was seen between the two fluids. This was due to the absence of the electro-elastic instability phenomenon at this low Weissenberg number. However, as the Weissenberg number increased to higher values, electro-elastic instability appeared within the geometry, which resulted in the mixing of two fluids, as shown in Figure 4b,c. The mixing intensity increased with an increasing value of the Weissenberg number. This is also evidenced in Figure 4e, wherein the mixing efficiency parameter η (in percentage values) is presented against the Weissenberg number values. One can see that as the Weissenberg number exceeded a critical value, the value of η increased drastically due to the appearance of electro-elastic instability. For instance, as the Weissenberg number increases from around 3 to 15, the value of η increases from around 30 to 60, so the increase in η is almost double. This efficient mixing inside this geometry resulted from a drastic change in the velocity field inside the geometry. In particular, a transition in the velocity field from steady and laminar to unsteady and irregular chaotic with different vortex structures was observed as the Weissenberg number gradually increased. This is shown in a phase diagram (Figure 4d) presented on a Wi−β space. In another study, Sasmal [64] showed that the mixing of two viscoelastic fluids can be obtained in a straight microchannel with variable wall zeta potentials utilizing the electro-elastic instability. This is demonstrated in Figure 4f, wherein the mixing behavior of two fluids (one dyed and another undyed) is shown again at three different Weissenberg numbers, namely, one, two, and three. The mixing was again not obtained at Wi=1 (first figure in Figure 4f); however, as the Weissenberg number increased to two (second figure in Figure 4b), mixing started, the intensity of which further increased as the Weissenberg number further increased to three (second figure in Figure 4b). Figure 4g shows this more quantitatively, wherein, once again, the mixing index parameter η is presented against the Weissenberg number. Once again, it increased once the Weissenberg number exceeded a critical value, as seen in the case of a long microchannel with step expansion and contraction. Ji et al. [65] conducted a numerical study on a two-dimensional microchannel with a constriction present in the middle of it. They also observed an unstable and spatially varying flow field once the applied electric field exceeded a critical value, likewise found in other studies, due to the EEI phenomenon.

**Figure 5:**
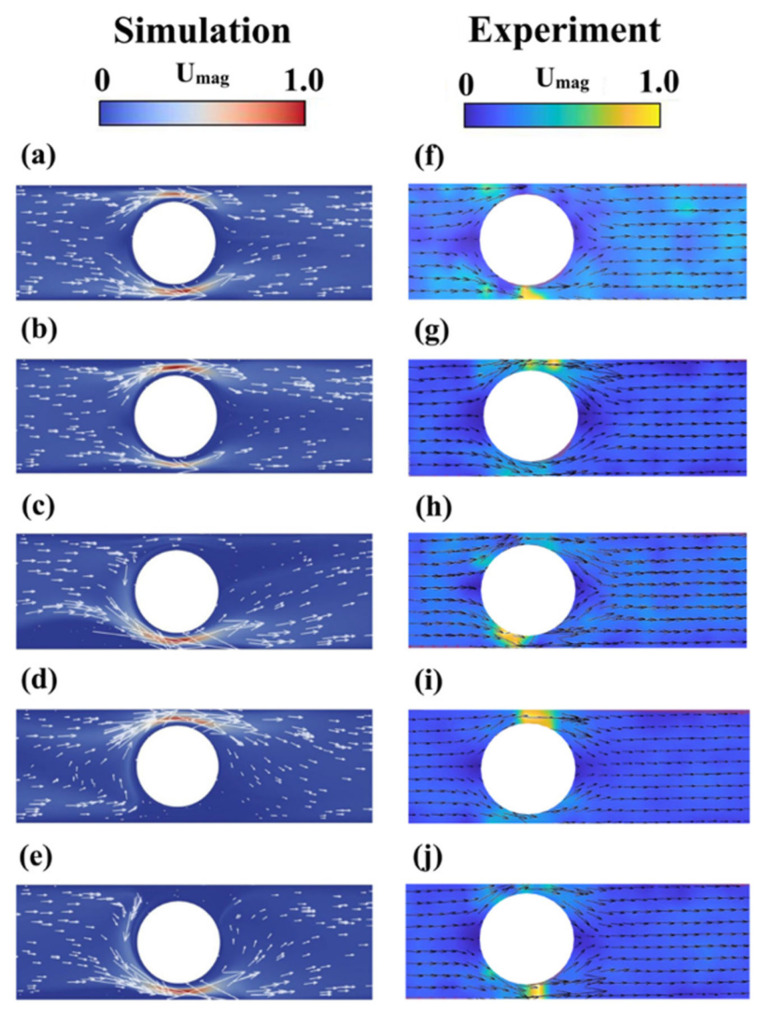
Experimental and numerical studies of electro-osmotic flows of viscoelastic fluids through a microchannel with a cylindrical obstacle present in it by Khan et al. [66]. The velocity magnitude and velocity vector plots at two different applied electric field strengths $\left(E_{x}\right)$, namely, 2857.14 V/m, 4285.71 V/m. For $E_{x}=2857.14$ V/m, results at two different times in the simulations (**a**,**b**) and for the experiments (**f**,**g**). For $E_{x}=4285.71$ V/m, results at three different times in simulations (**c**–**e**) and in experiments (**h**–**j**) are shown.

The electro-osmotic flows of viscoelastic fluids through a microchannel with a cylindrical obstacle present in it were investigated by Khan et al. [66] using both experiments and numerical simulations. They used a constant shear viscosity viscoelastic Boger fluid (comprising polyethylene oxide dissolved in polyethylene glycol and deionized water) in their experiments and the Oldroyd-B viscoelastic constitutive equation for their corresponding numerical simulations. Both the experiments and simulations were carried out for a wide range of applied electric field strengths ($E_{x}$). Figure 5 displays the velocity magnitude and velocity vector plots for different values of $E_{x}$ obtained both from the experiments and simulations. When the value of $E_{x}$ was 2857.14 V/m, both the experiments (see Figure 5a,b) and simulations (see Figure 5f,g) exhibited almost a steady and symmetric flow field within the geometry, as can be evidenced by the results presented at two different times. However, as the value of $E_{x}$ increased to 4285.71 V/m, the flow became highly time-dependent due to electro-elastic instability, as seen both in the experiments (Figure 5c,d) and simulations (Figure 5h–j) at three different times. Interestingly, it can be seen that most of the fluid flowed through the lower gap region between the cylinder and the channel surface at a time instance (Figure 5c,h in the simulations and experiments, respectively), and, in the next time instance, it occurred through the upper gap region (Figure 5h,i in the simulations and experiments, respectively). Therefore, a flow-switching phenomenon was observed inside the geometry once the applied electric field exceeded a critical value. Their numerical simulations also showed that this flow-switching phenomenon caused a rapid mixing of the two viscoelastic fluids placed in the upper and lower halves of the microchannel.

## 3. Conclusions and Future Directions

From the literature review in the preceding section, it is evident that electro-elastic instability leads to the transition of the flow field from a steady and symmetric state to an unsteady and asymmetric one during the electro-osmotic flow of viscoelastic fluids. This transition is strongly influenced by the critical Weissenberg number and/or the applied electric field (or voltage), which, in turn, depends on several factors, including the geometry of the system, polymer concentration, molecular weight, and the rheological properties of the viscoelastic fluid. While some studies have explored electrokinetically driven elastic instabilities, the research volume in this area remains limited compared to that on the corresponding pressure-driven or rotationally driven shear flows. This highlights a significant gap in the literature, offering considerable potential for further exploration, both for enhancing fundamental understanding and for expanding practical applications. In this discussion, we address these two key aspects of future research directions, emphasizing the need for deeper investigation into the mechanisms of electro-elastic instabilities and their potential, mainly in advanced microfluidic applications.

### 3.1. Scope in Fundamental Understanding

- In pressure-driven and rotationally driven shear flows, it is well established that when elastic instability arises, the flow field transitions to a more chaotic and fluctuating state, often leading to the onset of elastic turbulence as the fluid elasticity or flow strength increases [40,41]. However, for electro-osmotic flows, experimental investigations of electro-elastic turbulence remain largely limited in the literature. Although Pimenta and Alves [52] briefly mentioned this phenomenon in their study, they were unable to confirm its presence due to the absence of a detailed fixed-point data analysis of the fluctuating flow field. Thus, there is a significant need for further research into this regime. Future studies should include comprehensive statistical analyses of fixed-point fluctuating flow data and the temporal evolution of the entire flow field [67]. This can be achieved using advanced techniques such as micro-particle image velocimetry (μ-PIV) or other flow visualization methods, allowing for a more detailed characterization of the electro-elastic turbulence in electro-osmotic flows. Expanding the understanding of this regime could have important implications for enhancing transport processes in microfluidic applications. Along with experiments, further numerical simulations are needed to understand these EEI and EET phenomena better. However, the simulation of viscoelastic fluids remains a challenge due to the inherent “High Weissenberg Number Problem (HWNP)” associated with these fluids [68]. This problem leads to the loss of numerical stability at sufficiently high Weissenberg numbers, where EET flow is expected to exist. Some studies have used numerical stabilization techniques, such as the log-conformation tensor approach [69], and performed simulations in this regime [63,66]. More such stabilization techniques should be proposed to perform numerical simulations more efficiently at high Weissenberg numbers than possible with the currently available stabilization methods. However, simulations have been performed on these two phenomena so far using only the continuum approximation of the flow system. This approach cannot provide details on how fluid microstructure (such as polymer molecules in the case of polymer solutions) evolves in this EET regime, which could answer many questions related to this flow phenomenon. To do so, we must adopt a micro–macro- or multiscale simulation approach for viscoelastic fluids [70,71], which solves the governing equations of both the continuum solvent and dispersed phases, and, ultimately, solutions are coupled with each other through a proper algorithm. Although this simulation approach would provide more details, it will be more challenging to implement than the continuum approach. Therefore, a huge scope is present in this direction for future studies.
- A detailed investigation is required to determine whether significant differences exist in the evolution of the local and global flow fields over time or the onset mechanisms between electrokinetically driven and pressure-driven elastic instability and elastic turbulence phenomena. This question naturally arises because a significant difference exists in the flow dynamics between pressure- and electrokinetic-driven flows. For instance, the velocity field shows a parabolic profile in the former case, whereas a plug-like profile is seen in the latter case. This may cause different mechanisms for the onset of these instabilities for pressure- and electrokinetically driven flows. This clarification is very crucial as some experimental studies have suggested that EEI does not enhance mixing to the same degree as the elastic instability observed in pressure-driven or rotational shear flows [49,52]. A potential explanation for this difference lies in the variation in velocity profiles between the two flow types. Electrokinetically driven flows typically exhibit a plug-like velocity profile in microchannels [1], whereas pressure-driven flows tend to follow a parabolic velocity profile. These differing velocity distributions may lead to distinct flow behaviors once the EI and ET regimes are triggered. Therefore, further comparative studies, particularly focusing on temporal and spatial flow field characteristics in both these flow conditions, are necessary to understand the underlying dynamics and potential impacts on processes like mixing in a microscale system.
- When two non-Newtonian fluids with differing electrical conductivities flow under the influence of an applied electric field, the phenomenon of electrokinetic instability (EKI) often arises in microfluidic systems once the electric field strength and the conductivity gradient exceeds critical thresholds [72]. An intriguing area of research involves investigating the electro-elastic instabilities in two viscoelastic fluids, especially when these fluids also exhibit electrical conductivity gradients that could generate electrokinetic instabilities. A thorough statistical analysis of both the fixed-point fluctuating flow field data and the global flow field data is required to discern whether these two types of instabilities, electrokinetic and electro-elastic, behave similarly or if distinct differences exist between them. This investigation is crucial, as recent experimental and numerical studies have indicated that the viscoelastic properties of fluids tend to suppress the chaotic fluctuations associated with electrokinetic instability [73,74]. Understanding this suppression effect could have significant implications for controlling the instabilities in microfluidic systems.
- In the case of pressure-driven purely elastic instability and elastic turbulence phenomena, it has been observed that the shear-thinning properties (the apparent viscosity decreases with increasing deformation rate) of a viscoelastic fluid tend to suppress the chaotic behavior arising due to these instabilities and/or suppress the onset of these instabilities [75,76]. However, no detailed study has been performed on the corresponding electrokinetic-driven flows, emphasizing the influence of different non-linear rheological behaviors, such as shear-thinning or yield stress, on the onset of EEI and EET phenomena. Therefore, a great scope is present in this direction for further studies both in terms of experiments as well as numerical simulations.

### 3.2. Scope in Practical Applications

- Both electro-elastic instability and electro-elastic turbulence generate chaotic and fluctuating flow fields, which could enhance the mixing efficiency of two fluids in microfluidic systems. Chaotic flow patterns are known to significantly improve mixing, as demonstrated by several experimental and numerical studies investigating pressure-driven or rotationally driven elastic instabilities and elastic turbulence. These studies have shown a marked increase in mixing efficiency in viscoelastic fluids [43,44,45,54,77,78]. However, in the case of EEI and EET, the findings are less consistent. Numerical studies [63,64,66] suggest that these phenomena can lead to increased mixing efficiency, with significant improvements over Newtonian fluid behavior. On the other hand, some experimental studies [51,52] have reported either negligible increases or even decreases in mixing efficiency when EEI and EET are involved, particularly when compared to simple Newtonian fluids under similar conditions. These discrepancies between numerical and experimental results indicate that the mechanisms governing mixing in electro-elastic systems are not yet fully understood. To resolve these inconsistencies and clarify the role of EEI and EET in fluid mixing, further detailed studies, consisting of both experimental and numerical studies, are necessary. These investigations should focus on understanding the underlying flow dynamics and the conditions that lead to either enhancement in or suppression of mixing efficiency in microfluidic systems utilizing EEI and EET phenomena.
- Another promising application area deserving attention is microscale heat transfer through the utilization of electro-elastic instability and electro-elastic turbulence phenomena. Pressure-driven or rotationally driven elastic instability and elastic turbulence phenomena have already demonstrated significant enhancement in heat transfer rates in various microscale geometries when compared to simple Newtonian fluids under identical operating conditions [48,79,80,81,82,83,84,85]. The increased heat transfer efficiency results from the chaotic flow patterns generated by these instabilities, which lead to better heat distribution across the fluid. However, studies investigating microscale heat transfer using the corresponding EEI and EET phenomena are notably absent in the current literature. Exploring this area could provide valuable insights for enhancing the thermal performance of microscale systems, especially in applications where the precise control of heat transfer is critical. Potential applications include microfluidic heat exchangers, microfluidic cooling systems, microfluidic fuel cells, and microreactors. These systems frequently encounter heat transfer challenges due to their small dimensions and high heat generation rates. The introduction of EEI- and EET-based mechanisms could significantly improve thermal management by promoting enhanced convective heat transfer, thereby increasing the overall efficiency of these microscale devices. Further investigation into these phenomena could lead to new methods for optimizing the thermal performance of microfluidic systems, ultimately improving energy efficiency.
- Electrokinetic remediation (EKR) is an in situ environmental remediation technique that employs electrical currents to remove contaminants such as heavy metals, radioactive materials, and organic compounds from soils, sediments, or groundwater [8,86,87,88]. This process is driven primarily by electro-osmotic flow through porous media, which typically consist of soils or sediments. The introduction of electro-elastic instability and electro-elastic turbulence phenomena in these systems could enhance the contaminant removal efficiency, making this an area worthy of further investigation. This is particularly significant because studies on pressure-driven elastic instability and elastic turbulence in porous media have already demonstrated substantial improvements in oil displacement efficiency [89,90,91,92] and increased reaction rates [93], attributed to the chaotic and enhanced flow dynamics induced by these instabilities. Similar mechanisms could be leveraged in EKR, where the presence of EEI and EET may lead to more effective contaminant mobilization and transport through porous soils, improving overall remediation outcomes. Given the potential benefits, exploring the role of these elastic phenomena in EKR systems could open new avenues for optimizing contaminant removal, particularly in low-permeability soils where traditional methods struggle to achieve efficient remediation.

Finally, it should be highlighted that the practical application of EEI and EET phenomena for enhancing either microscale mixing or heat transfer rate depends on several factors, including the rheological properties of the fluids, operating conditions, and system geometry. While Boger fluids, characterized by constant shear viscosity and high extensional viscosity, are often used in studies due to their ability to leverage elastic turbulence [94] fully, their formulation requires the careful selection of polymers, concentrations, and solvents. In practical microfluidic applications, working fluids like polymer solutions, emulsions, suspensions, and biological fluids (e.g., blood, saliva, DNA, protein solutions, and cerebrospinal fluids) typically exhibit non-Newtonian behaviors such as shear-thinning, shear-thickening, viscoplasticity, and thixotropy [25]. These complex rheological properties can significantly influence EEI and EET dynamics and limit their effectiveness compared to Boger fluids. For heat transfer applications, engineered coolants mimicking Boger fluid behavior could exploit elastic turbulence, though high voltage differences may be required for pumping. In mixing applications, achieving the desired rheological properties poses greater challenges, as the experimenter may have limited control over the fluid composition. While adding polymers or surfactants can induce viscoelasticity, this does not guarantee Boger-like behavior due to various influencing factors. Additionally, such additives may be prohibited in certain applications due to downstream processing constraints. Therefore, a detailed understanding of fluid rheology is crucial for optimizing EEI and EET phenomena, particularly in scenarios involving non-Newtonian fluids.

Furthermore, the geometrical configuration of a system can significantly constrain the practical application of EEI and EET phenomena. Elastic instability arises from the interaction between normal elastic stresses and the curvature of streamlines [95,96], making curved geometries essential for its onset. However, designing and fabricating such geometries, particularly microscale ones, can be challenging. The type, arrangement, and design of these curved surfaces critically influence the onset and behavior of elastic turbulence, necessitating rigorous optimization studies, which have increased cost and complexity compared to simpler designs. Moreover, elastic turbulence inherently introduces chaotic flow fluctuations, which can pose challenges in applications requiring precise control, such as uniform mixing or chemical reactions. This randomness may result in non-uniform mixing rates and inconsistent outcomes, reducing the effectiveness of EEI and EET in processes demanding high accuracy and reproducibility. These challenges emphasize the importance of carefully considering both geometric design and flow dynamics when incorporating EEI and EET phenomena into practical systems and highlight the need for robust optimization and control strategies.

## Abbreviations

The following abbreviations are used in this manuscript:

EOF	Electro-osmotic flows
EEI	Electro-elastic instability
EET	Electro-elastic turbulence
EDL	Electrical double-layer
